# ﻿*Sinosasadamingshanensis* (Poaceae, Bambusoideae), a new combination supported by morphological and molecular evidence

**DOI:** 10.3897/phytokeys.255.148471

**Published:** 2025-04-08

**Authors:** Yi-Ting Zhang, Shu-Yan Lin, Zheng-Yang Niu, Wei-Xin Jiang, Nian-He Xia, Yu-Long Ding

**Affiliations:** 1 College of Life Sciences, Nanjing Forestry University, Nanjing 210037, China Nanjing Forestry University Nanjing China; 2 Key Laboratory of Plant Resources Conservation and Sustainable Utilization, Guangdong Provincial Key Laboratory of Digital Botanical Garden, South China Botanical Garden, Chinese Academy of Sciences, Guangzhou 510650, China South China Botanical Garden, Chinese Academy of Sciences Guangzhou China; 3 Guangxi Key Laboratory of Forest Ecology and Conservation, Key Laboratory of National Forestry and Grassland Administration on Cultivation of Fast-Growing Timber in Central South China, College of Forestry, Guangxi University, Nanning 530004, China Guangxi University Nanning China

**Keywords:** Arundinarieae, new combination, phylogeny, taxonomy

## Abstract

Morphological characteristics and phylogenetic analyses clearly revealed that *Chimonobambusadamingshanensis* should be a member of the genus *Sinosasa*, rather than *Chimonobambusa*, and is a distinct species close to *Sinosasahuapingensis* and *S.mingyueshanensis*. Morphologically, it differs from all the other known *Sinosasa* species by internodes initially with white pubescent, culm leaf auricles absent and triangularly subulate sheath blades, characteristics that are unusual in this genus. And this morphological distinction warrants recognition of *Ch.damingshanensis* as a new combination of *Sinosasa*.

## ﻿Introduction

*Sinosasa* L.C.Chia ex N.H.Xia, Q.M.Qin & Y.H.Tong (Poaceae, Bambusoideae), was first recognized as a genus of the temperate woody bamboo (i.e. Arundinarieae) by Q. M. Qin et al. (2021), and segregated from the genus *Sasa*Makino and Shibata (1901). It is characterized by racemose synflorescences, 3 stamens, 2 stigmas per floret, branches solitary and supranodal ridge strongly raised (Qin et al. 2021). So far, 8 species endemic to subtropical areas of China (Qin et al. 2021; Li et al. 2023), were included in the genus.

*Chimonobambusadamingshanensis* Hsueh & W. P. Zhang (1988) was described based on the only collection *C. J. Hsueh 8605* from Daming Mountain, Nanning, Guangxi Province, China. It is widely accepted in the flora accounts and monographs (Ohrnb 1990; Zhu et al. 1994; Hsueh and Wang 1996; Li and Stapleton 2006; Yi et al. 2008; Yi et al. 2009; Maria et al. 2018). In the protologue, it is described as “Rhizoma ampnipodiale; culmi graciles, erecti, caespitosi…; nodis culmorum plane protuberantibus, nodis vaginarum…basi 1-2 nodis radicibus aeriis…; Rami 1-3… Rami florum foliati; Ramuli florum basi 4-5-bracteati…”. All the important characters provided by the authors fit well with the circumscription of *Chimonobambusa*. After careful examination of the types (SWFC), we realized that the racemose synflorescence with real spikelets were misinterpreted as pseudospikelets and then the species *Ch.damingshanensis* is definitely not a member of the genus *Chimonobambusa*. In order to clarify the identity, we launched a special expedition to the type locality of the species, i.e. Daming Mountain, Nanning City, Guangxi Province of China, where only one bamboo with leptomorph rhizome, culm nodes prominent, culm sheath blades extremely small and foliage leaves with very long ligule, was found and it matches the protologue of *Ch.damingshanensis* very well. It is easily recognized as a member of *Sinosasa* rather than *Chimonobambusa* from branch compliment 1 per node, very prominent culm nodes and long ligule of foliage leaves. So, after comprehensive study, including morphological investigation, phenological observation, and phylogenetic reconstruction, *Chimonobambusadamingshanensis* was herein formally treated as a member of the genus *Sinosasa*, rather than *Chimonobambusa*.

## ﻿Materials and methods

The specimens of *Chimonobambusadamingshanensis* were obtained from field trips in 25 December of 2024. Its type specimen, *C. J. Hsueh 8605*, deposited in the Herbarium of Southwest Forestry University (SWFC), was examined. Some detailed characters, e.g. sheath ligule, were observed with hand lens and stereomicroscope (Leica S6D).

A total of 24 species representing 10 genera of the tribe Arundinarieae (Bambusoideae) were utilized to reconstruct a phylogeny based on complete chloroplast (cp) genome, among which *Bambusasinospinosa* McClure and *B.emeiensis* L. C. Chia & H. L. Fung were set as the outgroup taxa. All these sequences were previously published in GenBank. Accession numbers and voucher information are listed in Table 1.

**Table 1.** List of 24 bamboo taxa sampled in the present study with related voucher and GenBank accession information.

Taxon	Voucher information	Accession number
**Ingroup**		
*Acidosasapurpurea* (Hsueh & T.P. Yi) Keng f.	Zhang08023 (KUN)	HQ337793
*Ampelocalamusactinotrichus* (Merr. & Chun) S.L. Chen, T.H. Wen & G.Y. Sheng	MPF10003 (KUN)	MF066245
*Chimonobambusaangustifolia* C.D. Chu & C.S. Chao	Wu20210053 (YAFG)	OK040768
*Chimonobambusadamingshanensis* Hsueh & W. P. Zhang	WM241225 (NF)	PV021571
*Chimonobambusahejiangensis* C.D. Chu & C.S. Chao	GACP (NMGU)	MT884004
*Chimonobambusapurpurea* Hsueh & T.P. Yi	LW20200602-01 (CAAF)	MW030500
*Chimonobambusaquadrangularis* (Fenzi) Makino	CIMPC-RFM-20210302 (CMPC)	MW928533
*Chimonobambusasangzhiensis* (B.M. Yang) N.H. Xia & Z.Y. Niu	NZY109 (IBSC)	OM867788
*Chimonobambusatumidissinoda* Ohrnb.	MPF10083 (KUN)	MF066244
*Chimonobambusautilis* (Keng) Keng f.	Not provided by the author	OK040769
*Hsuehochloacalcareus* (C.D. Chu & C.S. Chao) D.Z. Li & Y.X. Zhang	MPF10050 (KUN)	KJ496369
*Indocalamussinicus* (Hance) Nakai	ZMY037 (KUN)	MF066250
*Indosasacrassiflora* McClure	BH58 (IBSC)	OK558536
*Indosasashibataeoides* McClure	MPF10028 (KUN)	MF066251
*Oligostachyumshiuyingianum* (L.C. Chia & But) G.H. Ye & Z.P. Wang	DZL09122 (KUN)	JX513423
*Pleioblastusamarus* (Keng) Keng f.	Zhang Yu-QuC373 (SANU)	MH988736
*Pleioblastusmaculatus* (McClure) C.D.Chu & C.S.Chao	MPF10161 (KUN)	JX513424
*Sasaveitchii* Rehder	LC1325 (ISC)	KU569975
*Sinosasafanjingshanensis* N.H. Xia, Q.M.Qin & J.B. Ni	BH124 (IBSC)	OP850348
*Sinosasagracilis* B.M.Yang	LX153 (IBSC)	OP973764
*Sinosasaguangxiensis* (C.D.Chu & C.S.Chao) N.H. Xia, Q.M. Qin & X.R. Zheng	CZY173 (IBSC)	OP850352
*Sinosasalongiligulata* (McClure) N.H. Xia, Q.M. Qin & J.B. Ni	CZY163 (IBSC)	OP850351
**Outgroup**		
*Bambusaemeiensis* L.C. Chia & H.L. Fung	Zhang08019 (KUN)	HQ337797
*Bambusasinospinosa* McClure	Li043 (KUN)	MK679807

By using the Plant Genomic DNA Kit (TSINGKE), total genomic DNA of *Ch.damingshanensis* was extracted from young and healthy leaves, and then sent to Novogene for DNA sequencing under the Illumina NovaSeq 6000 platform. A total of 40 G genome skimming data was used to assemble the complete chloroplast genome by GetOrganelle v 1.7.4 (Jin et al. 2018) using *Amborellatrichopoda* Baill. (accession number: NC_005086) and *Chimonobambusaluzhiensis* (J. R. Xue & T. P. Yi) T. H. Wen & Ohrnb. (accession number: NC_062708) set as the reference, with k-mer values of 21, 45, 65, 85, 105, 125 bp. The Bandage software (Wick et al. 2015) was employed to graphically visualize the assembled chloroplast. The complete chloroplast (cp) genome was annotated, and manually corrected in Geneious v9.1.4 (Kearse et al. 2012).

The matrix of all the whole chloroplast genomes was aligned in MAFFT v. 7.490 (Katoh and Standley 2013). Phylogenetic trees were constructed by using Maximum Likelihood (ML) and Bayesian Inference (BI). ML analysis was generated by IQ-TREE v.1.6.8 (Nguyen et al. 2015). BI analysis was generated by using MrBayes v 3.2.6 under the Akaike information criterion (AIC) (Ronquist et al. 2012). The best substitution model of TPM1uf was defined by jModeltest2 2.1.6 (Darriba et al. 2012).

Posterior Probability (PP) was obtained from Metropolis-coupled Markov Chain Monte Carlo (mcmc nruns = 2; ngen = 10,000,000; printfreq = 1,000; samplefreq = 1,000; nchains = 4; 25% burn-in).

## ﻿Results

**Figure 1.**
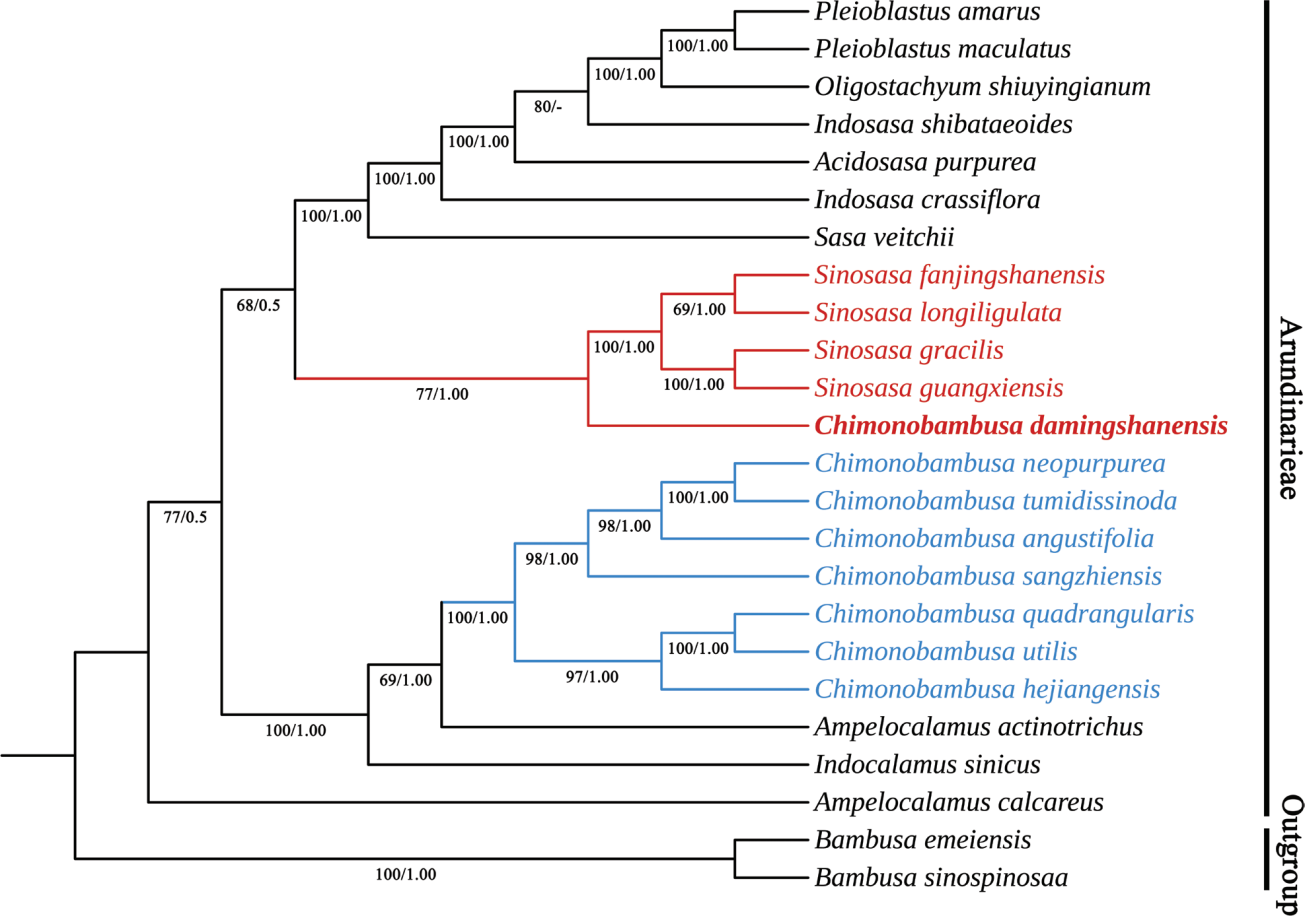
Phylogenetic relationships among *Chimonobambusadamingshanensis* and other 21species of Arundinarieae, based on plastid genome dataset with Maximum Likelihood and Bayesian analysis. Numbers above branches indicate maximum likelihood bootstrap support (BS) and posterior probabilities (PP), respectively.

The chloroplast genome size of *Chimonobambusadamingshanensis* is 139,964 bp and those of all the samples ranged from 139,394 bp (*Bambusamultiplex* L. C. Chia & H. L. Fung) to 140,013 bp (*Sinosasagracilis*) with an alignment of 159,910 bp. The phylogenetic tree topology generated by ML and BI analyses was somewhat congruent, differing only in the support values of the nodes, so only the ML tree was shown with nodal support values from both methods labelled (Fig. 1). As shown in the majority-rule consensus tree, *Chimonobambusadamingshanensis* is distantly related to other *Chimonobambusa* species, but forms a monophyletic clade with four *Sinosasa* species with nodal support (BS = 77% and PP = 1.00).

**Figure 2.**
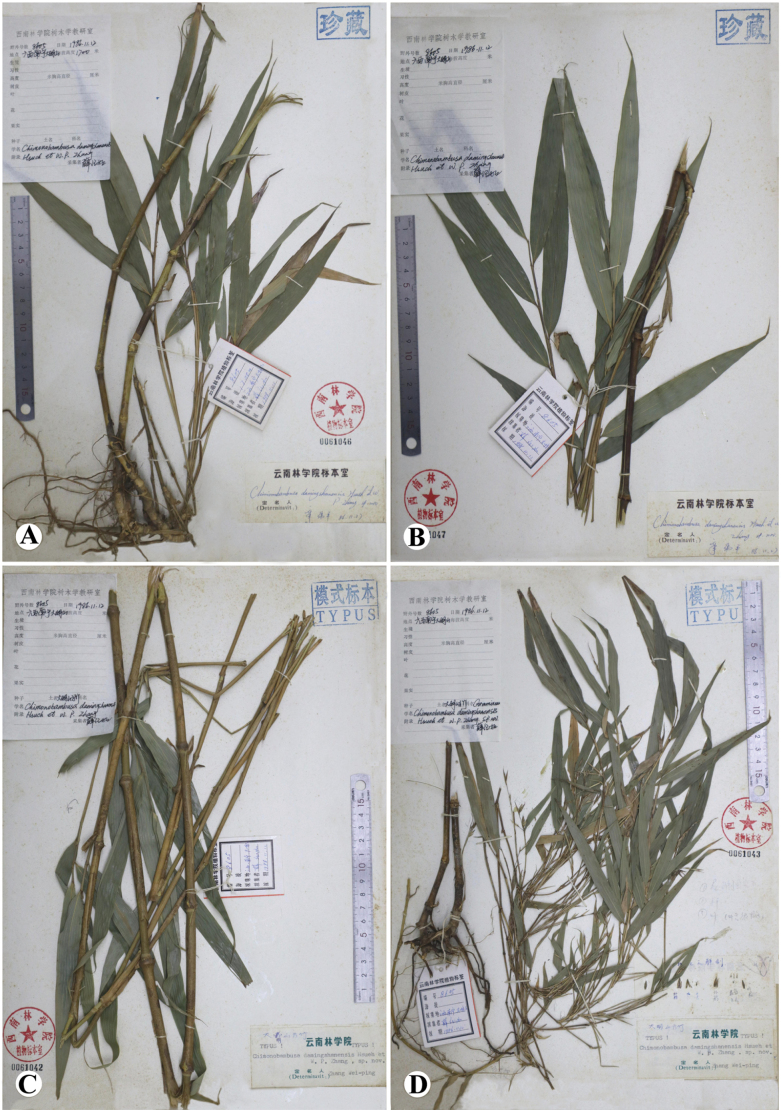
*Chimonobambusadamingshanensis* Hsueh & W. P. Zhang. **A**–**D***C.J. Hsueh 8605* (SWFC!). Photo by Yi-Ting Zhang.

**Figure 3.**
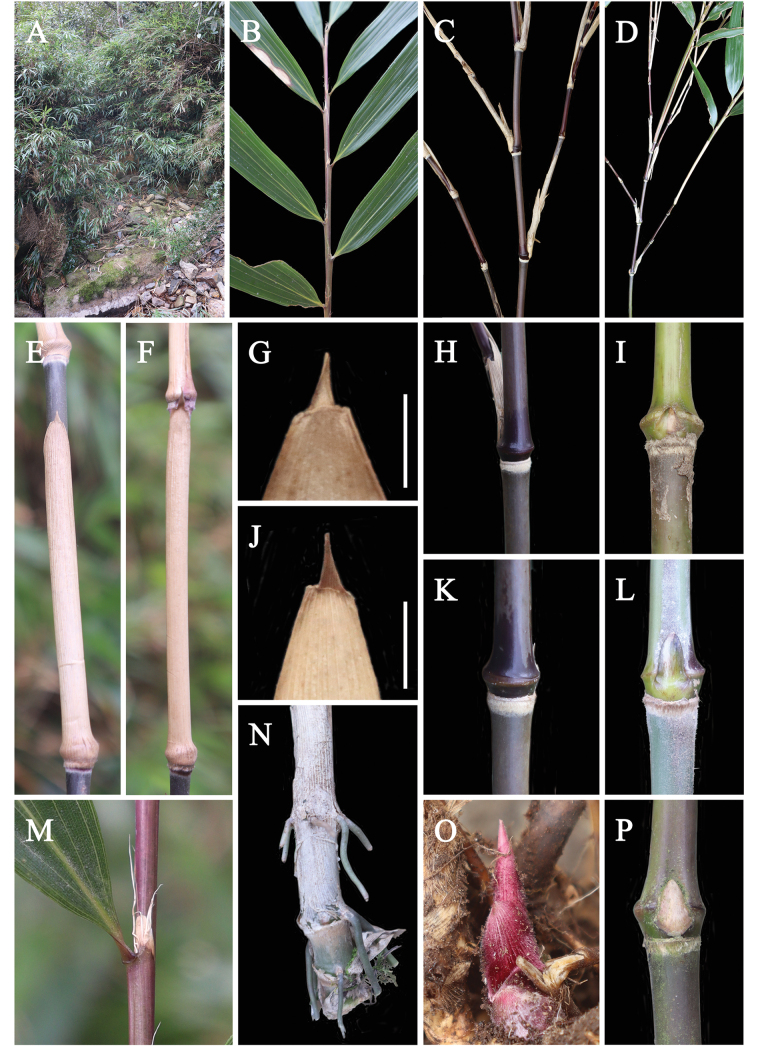
*Sinosasadamingshanensis***A** habitat and plants **B** foliage leaf branches **C** solitary branch **D** leafy branchlet and culm **E, F** culm and culm sheath **G** adaxial view of sheath blade **H** branch complements with a solitary primary axis **I, L, P** culm bud **J** abaxial view of sheath blade **K** culm node **M** leaf sheath, ligule and oral setae **N** basal culm nodes with root thorns (uncommon) **O** development of bamboo shoot under the ground. Scale bars: 1 cm (**G, J**).

Morphologically, *Chimonobambusadamingshanensis* resembles *Sinosasahuapingensis* N.H.Xia, Q.M. Qin & Y.H. Tong and *Sinosasamingyueshanensis* N.H.Xia, Q.M.Qin & X.R. Zheng in having branches solitary, culm leaf auricles absent, foliage leaf auricles and oral setae present (Figs 2, 3). However, it can be easily distinguished from the latter two species by the morphological characters (Table 2).

**Table 2.** Morphological comparison of *Chimonobambusadamingshanensis* and two related species.

Morphology	*Ch.damingshanensis*	*S.huapingensis*	*S.mingyueshanensis*
Young culm internodes	white pubescent gradually glabrous	sparsely brown hirsute gradually deciduous	upper part initially densely brown strigose, gradually glabrescent
Sheath blades	Triangularly subulate	Lanceolate	Lanceolate
Foliage leaf sheaths	Glabrous	Puberulent	Glabrous
Oral setae	4–8, 2.5–6 mm	2–4, 2–6 mm	4–6, 10–20 mm
Number of leaves on ultimate branch	5–10	8–17	6–7
Number of secondary veins of blades	3–4 (6) pairs	5–7 pairs	6–7 pairs
blades both surfaces	glabrous	adaxially glabrous, abaxially puberulent	glabrous

## ﻿Discussion

Our phylogenetic analysis demonstrated that *Sinosasa* is monophyletic (Fig. 1), consistent with the previous studies (Zeng et al. 2010; Guo et al. 2021). *Chimonobambusadamingshanensis* are described in the protologue as having 2 stigmas, and are observed in the type specimens as having true spikelets and 3 stamens. Molecular evidence from the plastid and morphological evidence further confirmed that *Chimonobambusadamingshanensis* should be a member of *Sinosasa* rather than *Chimonobambusa*, and thus a new combination in *Sinosasa* is proposed.

### ﻿Taxonomic treatment

#### ﻿ Sinosasa damingshanensis

Taxon classificationPlantaePoalesPoaceae

(Hsueh & W.P.Zhang) N.H.Xia & Y.L.Ding comb. nov.

559FD383-8756-5374-B153-4BAAB8F2DDDD

urn:lsid:ipni.org:names:77359971-1

Figs 2 , 3 , 4

##### Basionym.

*Chimonobambusadamingshanensis* Hsueh & W. P. Zhang, Bamb. Res. 7(3): 5. (1988).

##### Lectotype (here designated).

China • Guangxi, Nanning, Wuming, Daming Mountain, 12 Nov. 1986, *C. J. Hsueh 8605 fl.* (Lectotype: SWFC!).

##### Revised description.

Shrubby bamboos. Rhizomes leptomorph, rhizome internodes cylindrical, 2.45–4.75 cm long, nearly solid; nodes prominent, 2–4 roots at each node; rhizome bud ovate, ca. 4 mm high. Culms 1.5–2 m tall, 5–8 mm in diam; internodes terete, 5.4–13 cm long, initially with white pubescent gradually glabrous; supranodal ridge conspicuous, intranodes glabrous, 3–4 mm tall, infranodal region with a creamy-yellow and sericeous ring; branches solitary at each branching node. Culm bud solitary, triangular-ovate, sunken into supranodal ridge. Culm leaf sheaths persistent or tardily deciduous, papery, shorter than or as long as internode, abaxially glabrous or sparsely strigose, 6.8–13.3 cm high; sheath scar flat or slightly prominent; auricles and oral setae absent; blades erect, triangularly subulate, 1.6–4.5 mm high, glabrous. Foliage leaves 5–10 per ultimate branch; foliage leaf sheaths glabrous, purple or purple-green; auricles linear, 1–2 mm wide; oral setae erect or curled, 4–8, pale yellow, 2.5–6 mm long, easily deciduous when old; ligules developed, (3.3) 6–10 (–16) mm high, papery. Blades lanceolate, papery, 12.5–31.7 × 1.0–4.9 cm, both surfaces glabrous, margins serrulate along both sides, secondary veins 3–4 (6) pairs, significantly elevated on the lower leaf surface. The unit of the inflorescence raceme-like; lemma papery, ovate-lanceolate, ca. 10 mm long, glabrous; palea shorter than lemma, 6–7 mm long, 2-keeled; 2 stigmas, 3 stamens; ovary ellipsoid; style short. Fruit unknown.

**Figure 4.**
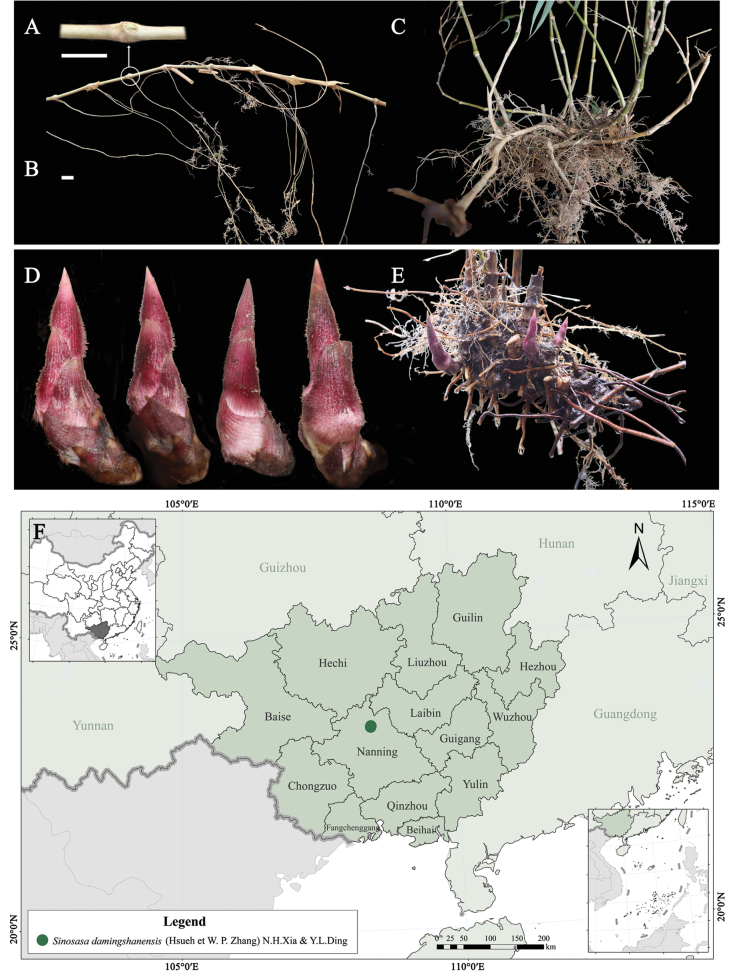
*Sinosasadamingshanensis***A** rhizome bud **B, C, E** rhizome **D** development of bamboo shoot under the ground **F** distribution map of *Sinosasadamingshanensis* in Nanning, Guangxi, China. Scale bars: 1 cm (**A, B**).

##### Notes.

*Chimonobambusadamingshanensis* Hsueh & W. P. Zhang was originally described based on *C.J. Hsueh 8605* which contains multiple specimens. According to the ICN (Turland et al. 2018) Art. 8.1, “The type (holotype, lectotype, or neotype) of a name of a species or infraspecific taxon is either a single specimen conserved in one herbarium or other collection or institution, or a published or unpublished illustration”, *C.J. Hsueh 8605 fl.* (SWFC, floriferous specimen) is designated here as the lectotype of *Ch.damingshanensis.* It has flower specimens and is more complete.

##### Phenology.

New shoots produced during April to May.

##### Chinese name.

大明山华赤竹(Chinese pronunciation: dà míng shān huá chì zhú).

##### Distribution and habitat.

Broad-leaved forests; It is endemic to Daming Mountain in Wuming District, Guangxi, China.

##### Additional specimen examined.

*Sinosasadamingshanensis*: China • Guangxi: Nanning, Wuming, Daming Mountain, 23°29′43.9″N, 108°26′14.0″E, alt. 1224–1445 m a.s.l., 25 December 2024, *Y. L. Ding & Y. T. Zhang WM241225* (NF!).

## Supplementary Material

XML Treatment for Sinosasa damingshanensis

## ﻿Additional information

### Author ORCIDs

Yi-Ting Zhang https://orcid.org/0000-0002-7068-9830

Shu-Yan Lin https://orcid.org/0000-0001-6326-2516

Zheng-Yang Niu https://orcid.org/0000-0003-0281-1504

Wei-Xin Jiang https://orcid.org/0000-0001-6447-8087

Nian-He Xia https://orcid.org/0000-0001-9852-7393

### Data availability

All of the data that support the findings of this study are available in the main text. The newly generated sequences were deposited in the GenBank database (accession numbers PV021571).

## References

[B1] Darriba D Taboada GL Doallo R Posada D (2012) jModelTest 2: More models, new heuristics and parallel computing. Nature Methods 9(8): 772. 10.1038/nmeth.2109 PMC4594756 22847109

[B2] Guo C Ma PF Yang GQ Ye XY Guo Y Liu JX Liu YL Eaton DAR Guo ZH Li DZ (2021) Parallel ddRAD and genome skimming analyses reveal a radiative and reticulate evolutionary history of the temperate bamboos. Systematic Biology 70(4): 756–773. 10.1093/sysbio/syaa076 33057686 PMC8208805

[B3] Hsueh CJ Wang ZP (1996) *Chimonobambusa.* In: Keng PC, Wang ZP (Eds) Flora Reipublicae Popularis Sinicae (Vol. 9). Science Press, Beijing, 324–344.

[B4] Hsueh CJ Zhang WP (1988) A Taxonomical Study of *Chimonobambusa* Makino, China. Bamboo Research 7(3): 5–7.

[B5] Jin JJ Yu WB Yang JB Song Y Yi TS Li DZ (2018) GetOrganelle: a simple and fast pipeline for de novo assembly of a complete circular chloroplast genome using genome skimming data. bioRxiv 256479. 10.1101/256479

[B6] Katoh K Standley DM (2013) MAFFT: multiple sequence alignment software version 7: improvements in performance and usability. Molecular Biology and Evolution 30(4): 772–780. 10.1093/molbev/mst010 23329690 PMC3603318

[B7] Kearse M Moir R Wilson A Stones-Havas S Cheung M Sturrock S Buxton S Cooper A Markowitz S Duran C Thierer T Ashton B Meintjes P Drummond A (2012) Geneious Basic: An integrated and extendable desktop software platform for the organization and analysis of sequence data. Bioinformatics (Oxford, England) 28(12): 1647–1649. 10.1093/bioinformatics/bts199 22543367 PMC3371832

[B8] Li DZ Stapleton CMA (2006) *Chimonobambusa* Makino. In: Wu ZY Raven PH Hong DY (Eds) Flora of China, vol.22. Science Press, Beijing, 152–161.

[B9] Li X Ni JB Cai ZY Tong YH Xia NH (2023) *Sinosasagracilis* (Poaceae, Bambusoideae), a new combination supported by morphological and phylogenetic evidence. PhytoKeys 226: 53–63. 10.3897/phytokeys.226.101164 37207081 PMC10189645

[B10] Makino T Shibata K (1901) On Sasa, a new genus of Bambuseae, and its affinities. Botanical Magazine Tokyo 15(168): 18–31. 10.15281/jplantres1887.15.168_18

[B11] Maria SV Lynn GC John D Rafaël G William JB (2018) World Checklist of Bamboos and Rattans. Science Press, Beijing, 41.

[B12] Nguyen LT Schmidt HA Haeseler V Minh BQ (2015) IQ-TREE: A fast and effective stochastic algorithm for estimating Maximum-Likelihood phylogenies. Molecular Biology and Evolution 32(1): 268–274. 10.1093/molbev/msu300 25371430 PMC4271533

[B13] Ohrnb D (1990) Gen. Chimonobambusa 42. The bamboo of the word: a preliminary study of the names and woody bamboos (Bambusoideae Nees v. Esenb.) documented in lists and maps. Langweid am Lech, 179 pp.

[B14] Qin QM Tong YH Zheng XR Ni JB Xia NH (2021) *Sinosasa* (Poaceae: Bambusoideae), a new genus from China. Taxon 70(1): 27–47. 10.1002/tax.12422

[B15] Ronquist F Teslenko M van der Mark P Ayres DL Darling A Hohna S Larget B Liu L Suchard MA Huelsenbeck JP (2012) MrBayes 3.2: Efficient bayesian phylogenetic inference and model choice across a large model space. Systematic Biology 61(3): 539–542. 10.1093/sysbio/sys029 22357727 PMC3329765

[B16] Turland NJ Wiersema JH Barrie FR Greuter W Hawksworth DL Herendeen PS Knapp S Kusber WH Li DZ Marhold K May TW McNeill J Monro AM Prado J Price MJ Smith GF [Eds] (2018) International Code of Nomenclature for algae, fungi, and plants (Shenzhen Code) adopted by the Nineteenth International Botanical Congress Shenzhen, China, July 2017. Regnum Vegetabile 159. Koeltz Botanical Books, Glashütten. 10.12705/Code.2018

[B17] Wick RR Schultz MB Justin Z Holt KE (2015) Bandage: Interactive visualization of de novo genome assemblies. Bioinformatics (Oxford, England) 31(20): 3350–3352. 10.1093/bioinformatics/btv383 26099265 PMC4595904

[B18] Yi TP Shi JY Ma LS Wang HT Yang L (2008) Illustrated Flora of Bambusoideae in China. Science Press, Beijing, 268.

[B19] Yi TP Ma LS Shi JY Wang HT Yang L Liu L (2009) Bamboo Subfamily Tribe and Genus Identification Key. Science Press, Beijing, 76.

[B20] Zeng CX Zhang YX Triplettd JK Yang JB Li DZ (2010) Large multi-locus plastid phylogeny of the tribe Arundinarieae (Poaceae: Bambusoideae) reveals ten major lineages and low rate of molecular divergence. Molecular Phylogenetics and Evolution 56(2): 821–839. 10.1016/j.ympev.2010.03.041 20381627

[B21] Zhu SL Ma NX Fu MY (1994) Iconographia bambusoidearum sinicarum. China Forestry Publishing House, Beijing, 158 pp.

